# Supplementary material for: Children and adolescents with overweight or obesity exhibit poor cardiorespiratory performance and elevated energy expenditure during an exercise task
Source: PLoS One. 2025 Jul 8;20(7):e0327875. doi: 10.1371/journal.pone.0327875 (PMC12237028; doi:10.1371/journal.pone.0327875)
Supplement: S1 Table — (DOCX) [file pone.0327875.s002.docx]

Supplementary Table 1: Cardiorespiratory fitness in children and adolescents grouped by nutritional state and sex.

|  | Healthy Weight | | Overweight | | Obesity | | Effect Size  ω² |
| --- | --- | --- | --- | --- | --- | --- | --- |
|  | ♀  n=45 | ♂  n=72 | ♀  n=36 | ♂  n=36 | ♀  n=21 | ♂  n=32 |  |
| V̇O_2_max  (ml·kg^-1^·min^-1^) | 35.0±5.9 | 38.8±6.1 | 32.7±5.2 | 35.9±5.3 | 27.6±6.3 | 32.8±5.8 | NS: 0.142  Sex: 0.08  Inter: unclear |
| V̇O_2_max at VT1  (ml·kg^-1^·min^-1^) | 23.91±3.97 | 25.34±5.31 | 22.59±3.95 | 23.6±5.53 | 20.39±4.23 | 23.29±3.67 | NS: 0.05  Sex: 0.03  Inter: unclear |
| %V̇O_2_max at VT1 | 68.9±8.72 | 67.35±10.61 | 67.8±12.3 | 65.95±12.64 | 74.8±9.69 | 71.5±8.26 | NS: 0.038  Sex: unclear  Inter: unclear |
| V̇O_2_max at VT2  (ml·kg^-1^·min^-1^) | 30.2±5.0 | 32.7±5.83 | 27.9±5.5 | 30.2±5.12 | 23.9±4.97 | 28.4±5.32 | NS: 0.111  Sex: 0.058  Inter: unclear |
| %V̇O_2_max at VT2 | 86.32±6.23 | 84.94±8.5 | 86.2±6.56 | 84.31±7.95 | 87.37±8.75 | 86.74±7.67 | NS: unclear  Sex: unclear  Inter: unclear |
