# Supplementary material for: Children and adolescents with overweight or obesity exhibit poor cardiorespiratory performance and elevated energy expenditure during an exercise task
Source: PLoS One. 2025 Jul 8;20(7):e0327875. doi: 10.1371/journal.pone.0327875 (PMC12237028; doi:10.1371/journal.pone.0327875)
Supplement: S2 Table — (DOCX) [file pone.0327875.s003.docx]

Supplementary Table 2: Cardiorespiratory fitness in children and adolescents grouped by nutritional state and sex.

|  | Low-CRF | | High-CRF | | Effect Size  ω² |
| --- | --- | --- | --- | --- | --- |
|  | ♀ | ♂ | ♀ | ♂ |  |
| V̇O_2_max at VT1  (ml·kg^-1^·min^-1^) | 20.4±3.79 | 21.97±3.73 | 25.7±3.5 | 26.3±5.23 | Sex: unclear  CRF: 1.0  Inter: unclear |
| %V̇O_2_max at VT1 | 72.0±11.26 | 71.1±10.66 | 66.3±8.42 | 64.2±12.09 | Sex: unclear  CRF: 0.069  Inter: unclear |
| V̇O_2_max at VT2  (ml·kg^-1^·min^-1^) | 24.7±4.23 | 33.1±3.08 | 26.9±3.46 | 34.2±5.17 | Sex: 0.018  CRF: 0.436  Inter: unclear |
| %V̇O_2_max at VT2 | 86.7±9.12 | 87.0±7.25 | 85.2±5.81 | 83.3±9.57 | Sex: unclear  CRF: 0.018  Inter: unclear |
