# Supplementary material for: Children and adolescents with overweight or obesity exhibit poor cardiorespiratory performance and elevated energy expenditure during an exercise task
Source: PLoS One. 2025 Jul 8;20(7):e0327875. doi: 10.1371/journal.pone.0327875 (PMC12237028; doi:10.1371/journal.pone.0327875)
Supplement: S3 Table — (DOCX) [file pone.0327875.s004.docx]

Supplementary Table 3: Cardiorespiratory performance, energy expenditure, rating of perceived exertion, and the post-effort recovery index grouped by nutritional status.

|  | Healthy Weight | | Overweight | | Obesity | | Effect Size  ω² |
| --- | --- | --- | --- | --- | --- | --- | --- |
|  | ♀  n=45 | ♂  n=72 | ♀  n=36 | ♂  n=36 | ♀  n=21 | ♂  n=32 |  |
| AUC  (arbitrary units) | 201.3±23.95 | 148.7±25.52 | 234.8±26.4 | 181.2±25.19 | 287.87±26.39 | 204.3±26.09 | - |
| V̇O_2_ in the exercise task  (ml·kg^-1^·min^-1^) | 25.63±3.25 | 25.97±3.64 | 25.7±4.62 | 25.45±3.67 | 22.98±4.13 | 25.51±4.1 | NS: 0.01  Sex: unclear  Inter: unclear |
| %V̇O_2_ in the exercise task | 72.94±12.64 | 68.2±11.98 | 74.73±12.31 | 71.75±10.1 | 83.89±10.22 | 78.37±9.48 | NS: 0.01  Sex: 0.033  Inter: unclear |
| RPE | 2.51±1.5 | 2.25±1.79 | 2.75±1.86 | 2.86±2.19 | 3.71±1.93 | 2.94±1.8 | NS: 0.03  Sex: unclear  Inter: unclear |
| Energy expenditure (kcal·min^-1^) | 5.63±1.31 | 6.12±1.75 | 6.88±1.68 | 7.35±1.87 | 8.2±2.3 | 7.66±2.29 | NS: 0.164  Sex: unclear  Inter: unclear |
| Ruffier index | 12.64±3.36 | 9.28±4.1 | 15.15±3.92 | 10.38±3.83 | 18.1±4.07 | 13.47±3.79 | NS: 0.147  Sex: 0.177  Inter: unclear |
| Dickson index | 10.06±4.21 | 6.47±3.19 | 12.31±4.81 | 8.17±3.19 | 16.25±5.08 | 10.21±4.67 | NS: 0.185  Sex: 0.141  Inter: unclear |
