# Supplementary material for: Children and adolescents with overweight or obesity exhibit poor cardiorespiratory performance and elevated energy expenditure during an exercise task
Source: PLoS One. 2025 Jul 8;20(7):e0327875. doi: 10.1371/journal.pone.0327875 (PMC12237028; doi:10.1371/journal.pone.0327875)
Supplement: S4 Table — (DOCX) [file pone.0327875.s005.docx]

Supplementary Table 4: Cardiorespiratory performance, energy expenditure, rating of perceived exertion, and the post-effort recovery index grouped by nutritional status.

| Girls | Healthy Weight | | Overweight | | | Obesity | | | Effect Size  η²p |
| --- | --- | --- | --- | --- | --- | --- | --- | --- | --- |
|  | VT1 | Exercise task | VT1 | Exercise task | | VT1 | | Exercise task |  |
| V̇O_2_  (ml·kg^-1^·min^-1^) | 23.91±3.97 | 25.23±4.2 | 22.13±4.77 | 25.3±5.15 | | 20.39±4.23 | | 22.98±4.13 | NS: 0.069  V̇O_2_: 0.2771  Inter: 0.051 |
| %V̇O_2_ | 68.88±8.7 | 72.94±12.64 | 67.76±12.3 | 74.73±12.31 | | 74.83±9.69 | | 83.89±10.22 | NS: 0.1092  V̇O_2_: 0.239  Inter: 0.031 |
| Boys | Healthy Weight | | Overweight | | | Obesity | | | Effect Size  η²p |
|  | VT1 | Exercise task | VT1 | | Exercise task | VT1 | Exercise task | |  |
| V̇O_2_  (ml·kg^-1^·min^-1^) | 25.34±5.31 | 26.22±4.2 | 23.6±5.53 | | 25.01±4.45 | 23.29±3.67 | 25.51±4.1 | | NS: 0.032  V̇O_2_: 0.079  Inter: 0.012 |
| %V̇O_2_ | 65.87±12.66 | 67.53±13.13 | 65.95±12.64 | | 70.4±12.72 | 71.54±8.26 | 78.37±948 | | NS: 0.092  V̇O_2_: 0.107  Inter: 0.028 |
