# Supplementary material for: Children and adolescents with overweight or obesity exhibit poor cardiorespiratory performance and elevated energy expenditure during an exercise task
Source: PLoS One. 2025 Jul 8;20(7):e0327875. doi: 10.1371/journal.pone.0327875 (PMC12237028; doi:10.1371/journal.pone.0327875)
Supplement: S5 Table — (DOCX) [file pone.0327875.s006.docx]

Supplementary Table 5: **Heart rate, energy expenditure, rating of perceived exertion, and the post-effort recovery index grouped by level of cardiorespiratory fitness.**

|  | Low-CRF | | High-CRF | | Effect Size  ω² |
| --- | --- | --- | --- | --- | --- |
|  | ♀ | ♂ | ♀ | ♂ |  |
| AUC  (arbitrary units) | 254.2±27.15 | 196.9±25.85 | 196.3±23.73 | 148.8±24.65 | - |
| V̇O_2_ in the exercise task  (ml·kg^-1^·min^-1^) | 23.34±4.08 | 24.41±3.44 | 26.96±3.27 | 26.77±4.52 | Sex: unclear  CRF: 0.106  Inter: unclear |
| %V̇O_2_ in the exercise task | 72.79±9.51 | 71.08±10.66 | 66.31±8.42 | 64.68±11.32 | Sex: unclear  CRF: 0.106  Inter: unclear |
| RPE | 3.26±1.81 | 2.8±2.02 | 2.22±1.53 | 2.38±1.83 | Sex: unclear  CRF: 0.033  Inter: unclear |
| Energy expenditure (kcal·min^-1^) | 6.79±2.13 | 7.06±2.07 | 6.32±1.59 | 6.58±1.99 | Sex: unclear  CRF: 0.035  Inter: unclear |
| Ruffier index | 16.19±4.03 | 12.49±3.96 | 12.36±3.42 | 9.0±3.89 | Sex: 0.138  CRF: 0.149  Inter: unclear |
| Dickson index | 13.74±5.25 | 9.44±4.07 | 9.74±3.91 | 6.43±3.26 | Sex: 0.144  CRF: 0.121  Inter: unclear |
