# Supplementary material for: Children and adolescents with overweight or obesity exhibit poor cardiorespiratory performance and elevated energy expenditure during an exercise task
Source: PLoS One. 2025 Jul 8;20(7):e0327875. doi: 10.1371/journal.pone.0327875 (PMC12237028; doi:10.1371/journal.pone.0327875)
Supplement: S6 Table — (DOCX) [file pone.0327875.s007.docx]

Supplementary Table 6: **Heart rate, energy expenditure, rating of perceived exertion, and the post-effort recovery index grouped by level of cardiorespiratory fitness.**

| Girls | Low-CRF | | High-CRF | | | Effect Size  η²p |
| --- | --- | --- | --- | --- | --- | --- |
|  | VT1 | Exercise task | VT1 | Exercise task | |  |
| V̇O_2_  (ml·kg^-1^·min^-1^) | 20.44±3.79 | 23.33±4.8 | 25.71±3.5 | 26.96±3.27 | | CRF: 0.28  V̇O_2_: 0.239  Inter: 0.047 |
| %V̇O_2_ | 72.0±11.26 | 80.14±13.03 | 66.31±8.42 | 69.71±9.32 | | CRF: 0.159  V̇O_2_: 0.202  Inter: 0.043 |
| Boys | Low-CRF | | High-CRF | | | Effect Size  η²p |
|  | VT1 | Exercise task | VT1 | | Exercise task |  |
| V̇O_2_  (ml·kg^-1^·min^-1^) | 21.97±3.73 | 24.41±3.44 | 26.32±5.23 | | 26.77±4.52 | CRF: 0.172  V̇O_2_: 0.085  Inter: 0.042 |
| %V̇O_2_ | 71.08±10.66 | 78.24±10.05 | 64.18±12.09 | | 65.3±12.09 | CRF: 0.207  V̇O_2_: 0.113  Inter: 0.066 |
