# Supplementary material for: Children and adolescents with overweight or obesity exhibit poor cardiorespiratory performance and elevated energy expenditure during an exercise task
Source: PLoS One. 2025 Jul 8;20(7):e0327875. doi: 10.1371/journal.pone.0327875 (PMC12237028; doi:10.1371/journal.pone.0327875)
Supplement: Attachment — Submitted filename: Response to reviewer.docx [file pone.0327875.s009.docx]

**Manuscript PONE-D-24-44802**

**Title: Children and adolescents with overweight or obesity exhibit poor cardiorespiratory performance and elevated energy expenditure during an exercise task.**

All the authors are deeply grateful for the comments and critical points raised by the reviewer because they contributed to improving the scientific contents of our study.

**Editor comments:**

1. When submitting your revision, we need you to address these additional requirements.

**Query 1:** We appreciated it. We have carefully revised our manuscript following the instructions.

2. We noticed you have some minor occurrence of overlapping text with the following previous publication(s), which needs to be addressed:

**Query 2:** The text was changed as requested.

3. Thank you for stating the following financial disclosure:

The study was funded by Agencia Nacional de Investigación y Desarrollo (ANID), FONIS SA19I0106.

Please state what role the funders took in the study. If the funders had no role, please state: "The funders had no role in study design, data collection and analysis, decision to publish, or preparation of the manuscript."

If this statement is not correct you must amend it as needed.

Please include this amended Role of Funder statement in your cover letter; we will change the online submission form on your behalf.

**Query 3:** The cover letter was modified as suggested.

4. Your ethics statement should only appear in the Methods section of your manuscript. If your ethics statement is written in any section besides the Methods, please delete it from any other section.

**Query 4:** The text was modified as suggested.

**Reviewer comments:**

**Reviewer 1**

General Comments:

This study titled “Children and adolescents with overweight or obesity exhibit poor cardiorespiratory performance and elevated energy expenditure during exercise tasks” was carried out with the aim of “to investigate cardiorespiratory performance and energy expenditure in response to an exercise task in children and adolescents”.

The study is interesting, but two important factors were not considered in the population in question: maturity and body composition.

1. Perhaps if the authors had considered maturity as a covariate in the analysis, they could have observed different results. The body composition measures could expand on the most recent discussions about the influence of different body tissues on physical fitness.

**Query 1:** Our research group appreciates the opportunity to clarify this point. Maturity and body composition are important covariates when analyzing physical performance-related outcomes [1]. For this reason, we stated in the last paragraph that this is one of the limitations of our study. Unfortunately, we have not conducted any surveys or tests to determine maturity level. We also did not have tools to assess body composition that could have improved our analyses.

Reference

1. BHAMMAR DM, ADAMS-HUET B, BABB TG. Quantification of Cardiorespiratory Fitness in Children with Obesity. Med Sci Sports Exerc. 2019;51:2243–50.

2. The authors could further explore the relationship between obesity and physical fitness. Could it be that obese children and adolescents with good cardiorespiratory fitness had better results in the exercise task and lower RPE? Subgroup analyses could have been explored.

**Query 2:** As suggested, we performed a new analysis to associate low or high cardiorespiratory fitness with exercise task and RPE. The results were added in the figure 2, a new figure 4, and discussed. We sincerely appreciate the reviewer's insightful comments and critiques, as they have significantly enhanced the scientific content of our study.

3. A complete revision of the English writing is necessary.

**Query 3:** We revised the English writing as suggested.

4. I suggest deleting the list of abbreviations and including them correctly in the text.

**Query 4:** We deleted as suggested.

**MINOR**

ABSTRACT:

Many acronyms have not been defined in the abstract (BMI, HW, Ow, Ob, RPE, EE, HR). Some are in the list of abbreviations, but I do not see the need for such a list. It would be best to indicate the full terms in the abstract and in the text the first time they appear.

Would not something like “were measured” be missing before “using a modified Balke protocol”? (Line 35).

**Query:** The text was modified as suggested.

INTRODUCTION:

The last sentence of the first paragraph is unnecessary (repetitive) (Lines 83-84). Repetition of terms and acronyms (once the acronym has been defined, keep going with it) (Lines 86, 89, 91, 92...).

Define the BMI acronym in the introduction and not in the methods (which is the first time it appears – line 91).

**Query:** The changes was made as suggested.

METHODS:

Define “WHO” (line 128).

Do not repeat the term maximal oxygen consumption in line 142 and revise this entire sentence - line 143 has a verb in the future tense.

Figure 1 shows that the maximum ergometric test was carried out after the exercise task. However, this is not made clear in the text, nor is it the recovery time between the two.

Correct “hearth rate” in the Figure 1.

**Query**: We appreciate your comment as it allows us to clarify this. To avoid this confusion, we have reorganized the order of the paragraphs and changed the text and figure as suggested.

RESULTS:

Replace Table I with Table 1 (line 186).

**Query**: The text was changed.

Table 1 shows no significant difference in DBP for any group. Therefore, the p-value should be >0.05.

**Query**: The text was changed.

The sentences in lines 193 to 195 can be deleted (they repeat information already contained in the methods).

**Query**: The sentence was deleted.

The central lines in figure 2C do not seem to indicate the averages mentioned in the text (lines 204-206).

**Query**: The figure 2C was modified. Figure 2C was presented as mean and was changed to median and scatter plot.

There are parts of methods in the results (lines 213 and 216).

**Query**: The sentences was deleted.

Properly quote the letters in Figure 3 (RPE is not B).

**Query**: The sentences was deleted

Revise the sentence related to Figure 3C (lines 218-223).

**Query:** We have discussed further on this result and made the following changes, which are reflected in Figure 3C and the text. First, we modified Figure 3C (replaced for Figure 3D-E) by removing the colors that delineated ventilatory thresholds based on the average for each threshold and group. Also, we modified the text. Second, we calculated the number and percentage of subjects below or above VT1 with each individual's relative oxygen consumption values. For example, if subject A had VT1 at 21.0 ml·kg^−1^·min^−1^ and the oxygen consumption required for the exercise task was 19.0 ml·kg^−1^·min^−1^, he was counted as an individual performing work below VT1 (low intensity). If his oxygen consumption required for the same subject in the exercise task would have been 24 ml·kg^−1^·min^−1^, he would be classified as an individual performing physical work above VT1 (moderate-high intensity). With this, we hope to clarify this doubt and be more precise in the results we want to show.

Revise Figure 3E - the text indicates that RPE was also higher for obese people compared to overweight people and it is not indicated in the figure.

**Query**: The sentences was modified

Review the last sentence of the results - Ruffier and Dickson appear twice in the two sentences. In fact, the authors could delete the last sentence.

**Query**: The sentences was deleted.

DISCUSSION

Replace PSE by RPE (line 239).

**Query:** The text was corrected as requested.

The second paragraph of the discussion includes studies with an adult population.

The discussion should deal with studies involving children and adolescents. There are several studies on physical fitness and health in this population.

The authors do not point out the alignment references commented on in the fourth sentence of the second paragraph (lines 246-249).

The entire second paragraph needs revision. There are too many loose sentences and no references to back up your assertions.

The discussion lacks a better foundation for the population in question - children and adolescents. Some interesting articles on the subject could have been cited (PMID: 34333829; PMID: 23767805; PMID: 18816947).

**Query:** We have considered all the recommendations and made amendments to the second paragraph.

What do the authors mean by “Besides, overweight children and adolescents carried more fat than those with a healthy weight. This aligns with the low levels of physical activity described in the pediatric population (32,33)”?

**Query:** The text was corrected as requested.

**Reviewer 2**

I reviewed the manuscript by Sepúlveda et al., which aimed to investigate cardiorespiratory performance and energy expenditure in response to an exercise task in children and adolescents. Their results showed a reduction in VO2max as a function of weight gain, which increased energy expenditure and the sensation of exertion. However, the manuscript presents some academic concerns related to the effect of sex and the metabolic demand of aerobic parameters. Presenting only values in %VO2max may generate a bias in the results because obese individuals present lower values, implying a greater relative location of VT1 and VT2 than the other groups. Therefore, these doubts need clarification.

Abstract

Line 43: present the meaning of the acronym EE.

**Query:** We welcome suggestions for improving the quality of our research. The text was corrected as suggested.

Materials and Methods

Study design

Did the study use eligibility criteria for participants? I recommend presenting the inclusion and exclusion criteria for participants.

**Query:** We added inclusion criteria in the study design.

Regarding the sample size, was any criterion used to determine it? The groups analyzed are very different and the number of participants by sex is not presented.

**Query:** Our work did not include a determination of sample size. We acknowledge that this is a limitation of our study. Additionally, we will provide the number of participants by sex.

Cardiorespiratory fitness

Lines 138 – 139: I recommend more clarity on whether the increment inclines 2% at each stage or increases the speed by 2% from the initial one.

**Query:** The text was corrected as requested.

Statistical analyses

I recommend inserting an analysis to compare the sexes and groups in each aerobic parameter (VT1, VT2, and VO2max) in ml kg-1 min-1.

**Query:** We added as suggested.

For the regression analyses, I recommend presenting which tests were used to verify the assumptions of normality, independence, and homoscedasticity of the residuals, as well as the test to verify influential residuals and leverage points.

**Query:** In the statistical analyses, we added strategies to determine the residuals' normality, independence, and homoscedasticity. We also reviewed the plotting of residues and identifying possible outliers.

Considering that increased body mass affects energy expenditure, I recommend verifying whether height influences the results since height (15 cm) is the same for all participants.

**Query:** As suggested, we performed a correlation between energy expenditure and height regardless sex. As expected, there is a positive association as we show in the graph below. This result was discussed in our manuscripts [2].

| Pearson r |  |
| --- | --- |
| r | 0,6415 |
| 95% confidence interval | 0,5606 to 0,7103 |
| R squared | 0,4115 |
|  |  |
| P value |  |
| P (two-tailed) | <0,0001 |
| P value summary | **** |
| Significant? (alpha = 0.05) | Yes |
| Are the slopes equal?  F = 0.2774. DFn = 1, DFd = 237  P=0.5989 | If the overall slopes were identical, there is a 59.89% chance of randomly choosing data points with slopes this different. You can conclude that the differences between the slopes arenot significant. |

Reference

2. Oliveira TMD de, Oliveira CC, Albuquerque VS, Santos MR, Fonseca DS, José A, et al. Performance, metabolic, hemodynamic, and perceived exertion in the six-minute step test at different heights in a healthy population of different age groups. Motriz: rev educ fis. 2021;27:e10210020520.

I recommend inserting an effect size analysis for each test used and an estimate of the power to verify type II errors.

**Query:** We added as suggested.

Line 179: I recommend using α < 0.05.

**Query:** We modified as suggested.

Results

I recommend presenting whether there were any adverse events in the participants during the tests.

**Query:** We added as suggested.

I recommend presenting all the results for the entire group (as they are) and by sex.

**Query:** We added analyses by sex as suggested.

I recommend presenting the test statistics (degrees of freedom, test value), effect size, and classification.

**Query:** We added in the results.

Discussion

I recommend including a discussion on the effect of sex and increased body mass on each of the aerobic parameters in the three groups analyzed.

**Query:** We added this topic in the discussion.

Table I: I recommend presenting the results by sex and presenting the values as mean ± SD, 95% confidence interval, or quartiles. I also suggest including the values of VT1 and VT2 in ml·kg-1·min-1.

**Query:** We added as suggested.

I recommend checking for differences in height between HW and Ob.

**Query:** We checked as suggested.

Figure 2: I recommend changing the markers to black, as this makes it difficult to visualize the mean marking. I also recommend this for Figure 3. For graphs that present individual values, I suggest presenting them as violin plots and the individual values should be attached as supplementary material.

**Query:** We modified some graphs, but we think that the scatter plot is more specific for visualizing the distribution of our results.
