# Supplementary material for: Children and adolescents with overweight or obesity exhibit poor cardiorespiratory performance and elevated energy expenditure during an exercise task
Source: PLoS One. 2025 Jul 8;20(7):e0327875. doi: 10.1371/journal.pone.0327875 (PMC12237028; doi:10.1371/journal.pone.0327875)
Supplement: Attachment — Submitted filename: Response to reviewer 2nd.docx [file pone.0327875.s010.docx]

**Manuscript PONE-D-24-44802**

**Title: Children and adolescents with overweight or obesity exhibit poor cardiorespiratory performance and elevated energy expenditure during an exercise task.**

All the authors are deeply grateful for the comments and critical points raised by the reviewer because they contributed to improving the scientific contents of our study.

**Journal Requirements:**

Please review your reference list to ensure that it is complete and correct. If you have cited papers that have been retracted, please include the rationale for doing so in the manuscript text, or remove these references and replace them with relevant current references. Any changes to the reference list should be mentioned in the rebuttal letter that accompanies your revised manuscript. If you need to cite a retracted article, indicate the article’s retracted status in the References list and also include a citation and full reference for the retraction notice.

**Query 1:** The references were checked as requested. As you pointed out, reference 24 in our manuscript had a correction in 2013. However, this correction was solely an editing error related to an author's name and does not pertain to any original content errors. I have attached an image of the correction.

24. Skinner JS, McLellan TM. The transition from aerobic to anaerobic metabolism. Res Q Exerc Sport. 1980;51: 234–248. doi:10.1080/02701367.1980.10609285

**Reviewer comments:**

**Reviewer 1**

GENERAL:
The quality of the figures is poor, and the captions are very confusing. The categories of each graph in figures 3 and 4 are not well understood. The figures need to be understood without having to resort to the text. The authors should change the color of the legend in figure 2, as different categories should not appear in the same color within the same figure.

**Query 1:** We implemented the suggested changes.

ABSTRACT:
Define the abbreviation (CRF) after the full term the first time it appears.

**Query 2:** The text was changed as suggested.

METHODS:
I - Define the acronym for respiratory compensation point (RCP) (line 181) and replace the full term with the acronym in all other occurrences in the text (line 455, for example).

**Query 3:** The text was changed as suggested.

II - The authors report the variable subjective perception of effort (RPE) in the results and discussion, but they did not mention this variable in the methods and how it was measured.

**Query 4:** In the subheading "Exercise task" we describe the RPE registry with the following text: “At the end of the exercise task, the participants rated their perceived exertion using a modified Borg scale ranging from 0 to 10”.

RESULTS:

I - Table 1 is a bit confusing. It is not clear where the differences indicated by ANOVA are for the significant variables. This is also not described in the first paragraph of the results.

**Query 5:** We recognize that this may be confusing, so we have included a brief explanation of the table.

II - Replace the sentence “Girls and boys belong to Ow and Ob group had a higher heart rate than HW in the exercise task (Fig 3A-C)” by “Girls and boys belonging to the Ow and Ob groups had a higher heart rate than the HWs throughout the exercise task (Fig. 3A-C)” and add the overall p-value of these differences. (Lines 281-282).

**Query 5:** The text was changed as suggested.

III - There are still descriptions of methods in the results (pag. 295-296).

**Query 6:** The text was corrected as suggested.

IV - I believe that the authors were mistaken in the following answer:
Query: We have discussed further on this result and made the following changes, which are reflected in Figure 3C and the text. First, we modified Figure 3C (replaced for Figure 3D-E) by removing the colors that delineated ventilatory thresholds based on the average for each threshold and group. Also, we modified the text. Second, we calculated the number and percentage of subjects below or above VT1 with each individual&apos;s relative oxygen consumption values. For example, if subject A had VT1 at 21.0 ml·kg−1·min−1 and the oxygen consumption required for the exercise task was 19.0 ml·kg−1·min−1, he was counted as an individual performing work below VT1 (low intensity). If his oxygen consumption required for the same subject in the exercise task would have been 24 ml·kg−1·min−1, he would be classified as an individual performing physical work above VT1 (moderate-high intensity). With this, we hope to clarify this doubt and be more precise in the results we want to show.
The value of 21.0 ml-kg-1-min-1 is also higher than 19.0 ml-kg-1-min-1, as is 24 ml-kg-1-min-1. The text described here by the authors (except for the example) should be included in the methods.

**Query 7:** We apologize for this confusion. Each study participant counted whether oxygen consumption was above or below their VT1 in the exercise task. We will add an explanatory text in methods.

V - The author’s statement in lines 325-327 is only shown for girls in the graphs in Figure 3O-P. Revise.

**Query 8:** The text was corrected as suggested.

VI - Replace “Fig 3F-G” by “Fig 4F-G” (line 361).

**Query 9:** The text was corrected as suggested.

VII - Replace “Fig 3H-I” by “Fig 4H-I” (line 366).

**Query 10:** The text was corrected as suggested.

DISCUSSION
Describe the full term for FCR and eliminate the abbreviation (line 463).

**Query 11:** We apologize for this error. The abbreviation FCR is incorrect; the correct abbreviation is CRF. The text was revised and modified as suggested.

**Reviewer 2**

I suggest using the combining dot above also for ventilation (VE).

**Query 1:** The text was corrected as suggested.

Lines 190 – 191: I recommend presenting the low and high CRF classification criteria based on the z-score.

**Query 2:** We added a phrase as suggested.

Line 198: I suggest to use η2 as partial η2 (η2p), because η2 is an efficient way to compare effect sizes within a study, and cannot be easily compared between studies, because the total variability in a study (SStotal) depends on the design of a study and increases when additional variables are manipulated. As for η2p, it expresses the sum of the squares of the effect concerning the sum of the squares of the effect and the sum of the squares of the error associated with the effect.

**Query 3:** The analyses have been carefully reviewed and adjusted according to the suggestions provided. It is important to highlight that the values of η² and partial η² are nearly identical, which indicates there is no significant overlap in the variance among the effects in this ANOVA. This finding suggests that the effects are independent and do not compete with each other to explain the same.

I recommend presenting the CRF results by group and sex in tables and figures, this makes it easier to understand compared to the text.

**Query 4:** We added tables as supplementary material.
